# Modelling Growth-Competition Relationships in Trembling Aspen and White Spruce Mixed Boreal Forests of Western Canada

**DOI:** 10.1371/journal.pone.0077607

**Published:** 2013-10-24

**Authors:** Jian-Guo Huang, Kenneth J. Stadt, Andria Dawson, Philip G. Comeau

**Affiliations:** 1 Department of Renewable Resources, University of Alberta, Edmonton, Alberta, Canada; 2 Forest Management Branch, Sustainable Resource Development, Edmonton, Alberta, Canada; Helmholtz Centre for Environmental Research - UFZ, Germany

## Abstract

We examined the effect of competition on stem growth of *Picea glauca* and *Populus tremuloides* in boreal mixedwood stands during the stem exclusion stage. We combined traditional approaches of collecting competition data with dendrochronology to provide retrospective measurements of stem diameter growth. Several competition indices including stand basal area (BA), the sum of stem diameter at breast height (SDBH), and density (N) for the broadleaf and coniferous species, as well as similar indices considering only trees with diameters greater than each subject (BAGR, SDBHGR, and NGR), were evaluated. We used a nonlinear mixed model to characterize the basal area increment over the past 5, 10, 15, 20, 25, 30, and 35 years as a function of growth of nearby dominant trees, the size of the subject trees, deciduous and coniferous competition indices, and ecoregions. SDBHGR and BAGR were better predictors for spruce, and SDBHGR and NGR were better for aspen, respectively, than other indices. Results showed strongest correlations with long-term stem growth, as the best models integrated growth for 10–25 years for aspen and ≥25 for spruce. Our model demonstrated a remarkable capability (adjusted R^2^>0.67) to represent this complex variation in growth as a function of site, size and competition.

## Introduction

Both inter- and intraspecific competition play important roles in affecting forest growth, composition, structure, and succession. Following a stand-replacing disturbance, boreal mixedwood sites are typically dominated by shade-intolerant species such as trembling aspen (*Populus tremuloides* Michx.), white birch (*Betula papyrifera* Marsh.) and lodgepole or jack pine (*Pinus contorta* Dougl. ex. Loud. and *Pinus banksiana* Lamb.). Shade-tolerant white spruce [*Picea glauca* (Moench.) Voss] may co-establish with these pioneer species or establish later; these spruce grow steadily in the understory, reaching the canopy after 50–90 years [1], [2]. Late-successional balsam fir [*Abies balsamea* (L.) Mill.] establishes and emerges last, if fire does not occur [3], [4]. In Canada, mixedwood stands composed of trembling aspen and white spruce are a major component of the boreal forest, especially in the prairie provinces of western Canada [5]. In these mixedwood forests, competition is particularly critical during the stem exclusion or self-thinning stage (typically 20−40 years of stand age; [6]) and during the subsequent understory reinitiation stage. In these stages, critical transitions occur, such as crown separation of the pioneer species following self-thinning episodes, or spruce crown closure in stands with higher conifer density [4], [7]. Intense competition among trees for resources such as light, soil moisture and nutrients occurs as they expand in size, leading to logarithmic decreases in stem density [4], [8]. Spruce shows a strong response in radial and height growth to increases in light [9], [10]. In the boreal mixedwood forest of western Canada, poor conifer height growth under dense hardwood canopies was attributed to light below 20% of full sunlight, well below levels needed for optimum growth [9], [11]. Hence it is particularly critical to quantify the effects of competition on growth at this stage, to help us better understand forest growth and dynamics, and to improve forest management decisions. At a stand level, the emergent property of these tree dynamics may account for greater volume yields in mixed vs. pure stands [12], [13].

Previous studies of the links between competition and growth typically rely on permanent sample plot (PSP) data which consists of periodic re-measurements at 2–10 year intervals (e.g., breast height diameter, height, and conditions of trees) [14], [15], [16], [17]. PSP programs vary by jurisdiction and are often not well-structured in terms of age, productivity and composition. In the western boreal, data are particularly lacking for mixed species stands aged 30−70 years. The sparse nature of existing mid-rotation PSP data makes it difficult to confidently address questions regarding growth dynamics during the stem exclusion and understory re-initiation stages.

A dendrochronological approach may be more effective than PSPs to address this lack of data. Tree-ring analysis provides growth data without re-measurement of plots, and is therefore more efficient than PSP data [15]. Dendrochronology relies on inter-annual ring-width growth patterns to model tree growth dynamics as well as endogenous (e.g. competition) and exogenous (e.g., disturbance, climate) factors [18]. For example, Biondi [19] demonstrated how dendrochronological data can be used to obtain insight into stand growth patterns over longer periods than is possible using PSPs. Metsaranta and Lieffers [15] compared dendrochronological stand reconstruction techniques with boreal pine PSPs and found these techniques can provide similar information on stand development patterns, though dendrochronology provided annual resolution that is rarely obtained from PSP data. Numerous other studies [10], [20] have also used short sequences of the most recent growth rings to characterize growth rates in response to current competition.

To quantify the relationship between growth and competition during the stem exclusion and understory reinitiation period, we surveyed 51 stands in the boreal mixedwood region of Alberta using an efficient sampling technique which combined traditional approaches of collecting competition data with dendrochronology. The specific objectives of the study were to 1) model the relationships between stem growth, tree size and competition in the boreal mixedwood forest over different time intervals and ecoregions; 2) develop a sub-model to estimate the growth potential of dominant trees, and 3) identify competition indices suitable for long-term growth prediction of sub-dominants. We hypothesize that the growth response to competition can be more effectively predicted when conditioned by the growth of nearby dominant trees, that the pattern of growth rate vs. tree size can be simply modeled, and if competition effects are carefully characterized, provide a powerful model for predicting tree growth.

## Materials and Methods

### Ethics Statement

No specific permissions were required to conduct field research in our study areas; these are public forests, where scientific research is encouraged. We confirm that our field studies did not involve endangered or protected species.

### Study Area

**Figure 1:**
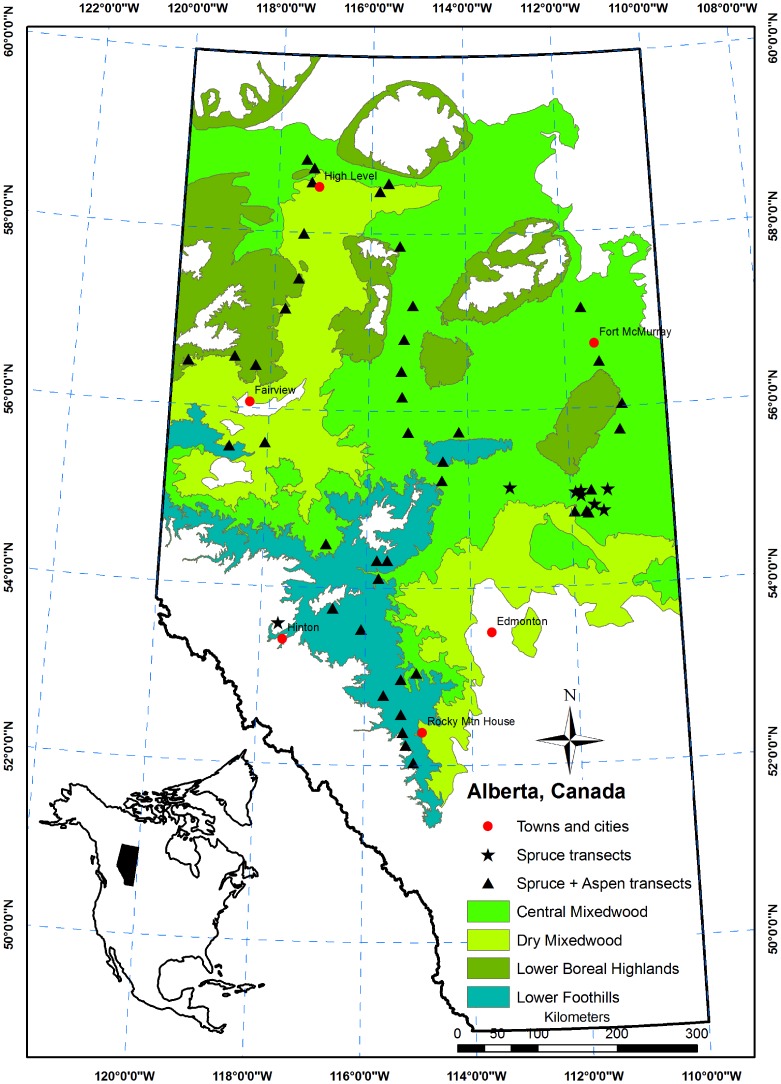
Map showing the locations of transects in relation to the natural ecoregions of Alberta, Canada.

Our study area included the mixedwood ecoregions in Alberta, Canada, which occupy 75% of the forested area of the province (Fig. 1). The upland forests in these regions are dominated by even- or uneven-aged mixtures of trembling aspen, white spruce, balsam poplar (*Populus balsamifera* L.), paper birch, and balsam fir [21], [22]. Lodgepole pine also occurs within these mixtures on the eastern slopes of the Rocky Mountains. Our sampling covered most of these regions, from Rocky Mountain House in the south to High Level in the north, and east from Fort McMurray to Fairview and Hinton in the west (Fig. 1). Sampling locations were distributed throughout the Central Mixedwood, Dry Mixedwood, Lower Foothills, and Lower Boreal Highlands ecoregions [23], [24], which contain the majority of the productive mixedwoods. Elevations of the sampled sites ranged from 266 m to 1308 m.

The region is dominated by a typically dry continental climate, with cold winters and warm summers. Mean annual temperature decreases with increasing latitude and elevation (i.e. northward and westward); precipitation increases with elevation. The 1971−2000 climate normals indicate mean annual temperatures, growing degree days (GDD>5°C), and mean annual total precipitation were 0.7°C, 1376.4°C, and 455 mm with 25% in the form of snow, respectively, in the easternmost portion of the study area [Fort McMurray, 56°39′N, 111°13′W, 369 m above sea level (a.s.l.)], and 2.1°C, 1377.5°C, and 471.6 mm with 30% in the form of snow, respectively, for the westernmost study region (Fairview, 56°04′N, 118°23′W, 670 m a.s.l.). At the southern extent of the study area (Rocky Mountain House, 52°26′N, 114°55′W, 988 m a.s.l.), these climate variables were 2.3°C, 1163.6°C, and 535.4 mm with 30% in the form of snow, respectively, and −1.3°C, 1225.7°C, and 394.1 mm (34% as snow) in the northern area (High Level, 58°37′N, 117°10′W, 338 m a.s.l.) [25]. Soils in these regions were mainly orthic gray luvisols and brunisols with primarily silty or clay loam texture, due to glacial till and glaciolacustrine parent material [23], [24].

### Data Collection

Field sampling was conducted in 2007, 2008, 2010 and 2011, and was designed to obtain data for this project as well as a retrospective white spruce mortality study (Dawson et al. in prep.). We randomly sampled accessible trembling aspen and white spruce dominated mixedwood stands, which ranged from 25 to 100 years according to the Phase 3 inventory database [26]. We excluded stands where the aspen cohort was clearly uneven-aged or stands with indications of ground fires or insect outbreaks. In each stand, a belt transect was employed to assess growth-competition relationships for trembling aspen and/or white spruce vs competitors. Transects ranged from 5 m to 785 m long and 5 m to 20 m wide. The transect area ranged from 25 to 1600 m^2^ (mean = 415 m^2^), and depended primarily on spruce density (low numbers of spruce required larger areas to obtain an adequate sample). Within each transect, all trees over 1 m tall were counted by species and classified into three size classes [1−3 m height (H), 3 m H −10 cm stem diameter at breast height (DBH; defined as 1.3 m above-ground), and over 10 cm DBH]. Eight to 15 live spruce and ten aspen were then randomly chosen in each transect as subject trees. Height, DBH, and a competition assessment were taken before each subject tree was felled and a disk of each subject tree was taken at stump height (0.3 m). For competitor assessment, the DBH of all trees and shrubs taller than 1.3 m and within a 1.78 m radius plot (10 m^2^) centered at the subject tree were measured. These local competition plots were then aggregated to obtain a transect-level assessment of competition for each species (usually>100 m^2^ of each transect’s area was sampled for competitors) during calculation of competition indices. Larger plots would be desirable for local neighbourhood competition assessments at each tree, but forest growth models are typically applied at the stand (transect) level [27]; this sampling was designed to assess competition at a similar scale.

We also sampled approximately ten of the largest aspen and five of the largest white spruce trees near the transect in the same ecosite to provide a measure of potential growth on the site. At most sites, these largest aspen and spruce were canopy dominants. However, at some sites, there were no dominant spruce present. In these cases, large spruce trees were still sampled, and their competition was assessed. The DBH of each dominant or large spruce tree was measured and two 5.1 mm increment cores from each of these trees were collected at 1.3 m height for annual ring-width measurement in the lab. Ecosite classification was performed for each transect, following Beckingham and Archibald [24] and Beckingham et al. [23]. In total, 51 transects were sampled for spruce growth-competition assessment; 42 of these were also sampled for aspen growth-competition assessment, though all were mixed stands (Fig. 1). In total, stem growth and competition was measured for 1670 trees (spruce: 620 subject trees and 227 dominant or large spruce trees; aspen: 420 subject trees and 403 dominant trees) throughout the mixedwood forests of Alberta (Table 1).

**Table 1:** Characteristics of the transects sampled in Alberta mixedwood forest, western Canada.

Natural subregion	Transects	Spruce																	
		Deciduous competition																	
		BA (m^2^/ha)			SDBH (m/ha)			N (stems/ha)			BAGR (m^2^/ha)			SDBHGR (m/ha)			NGR (stems/ha)		
		Max	Mean	Min	Max	Mean	Min	Max	Mean	Min	Max	Mean	Min	Max	Mean	Min	Max	Mean	Min
CM+DM	34	49	20	2	394	187	12	7600	1955	67	49	15	0	361	128	0	2600	1064	0
LF+LBH	17	28	15	4	332	159	43	19692	2738	400	28	12	3	206	110	31	8385	1282	250
		**Spruce competition**																	
		**BA (m^2^/ha)**			**SDBH (m/ha)**			**N (stems/ha)**			**BAGR (m^2^/ha)**			**SDBHGR (m/ha)**			**NGR (stems/ha)**		
		**Max**	**Mean**	**Min**	**Max**	**Mean**	**Min**	**Max**	**Mean**	**Min**	**Max**	**Mean**	**Min**	**Max**	**Mean**	**Min**	**Max**	**Mean**	**Min**
CM+DM	34	81	26	5	548	249	98	11500	3342	917	24	7	0	228	64	0	4600	683	0
LF+LBH	17	49	23	4	471	271	89	8400	4100	1133	20	7	0	135	66	2	2308	664	67
**Natural** **subregion**	**Transects**	**Aspen**																	
		**Deciduous competition**																	
		**BA (m^2^/ha)**			**SDBH (m/ha)**			**N (stems/ha)**			**BAGR (m^2^/ha)**			**SDBHGR (m/ha)**			**NGR (stems/ha)**		
		**Max**	**Mean**	**Min**	**Max**	**Mean**	**Min**	**Max**	**Mean**	**Min**	**Max**	**Mean**	**Min**	**Max**	**Mean**	**Min**	**Max**	**Mean**	**Min**
CM+DM	26	49	24	9	423	245	82	7600	2726	643	45	13	0	362	101	0	3000	717	0
LF+LBH	16	28	17	4	308	173	43	4867	2080	400	28	11	0	264	80	0	2133	529	0
		**Spruce competition**																	
		**BA (m^2^/ha)**			**SDBH (m/ha)**			**N (stems/ha)**			**BAGR (m^2^/ha)**			**SDBHGR (m/ha)**			**NGR (stems/ha)**		
		**Max**	**Mean**	**Min**	**Max**	**Mean**	**Min**	**Max**	**Mean**	**Min**	**Max**	**Mean**	**Min**	**Max**	**Mean**	**Min**	**Max**	**Mean**	**Min**
CM+DM	26	48	25	9	610	305	108	13000	4495	1438	36	12	0	369	92	0	3750	650	0
LF+LBH	16	49	23	5	392	262	97	8500	3868	1200	40	11	0	276	77	0	2125	478	0

Abbreviations: CM+DM: Central Mixed wood and Dry Mixed wood; LF + LBH: Lower Foothills and Lower Boreal Highlands; BA: sum of basal area; SDBH: sum of diameter at breast height; N: density; BAGR: sum of basal area for trees thicker than each subject; SDBHGR: sum of diameter at breast height for trees thicker than each subject; NGR: density for trees thicker than each subject.

In the laboratory, all tree-ring samples (discs and cores) were dried and carefully polished with successively finer grits of sandpaper. The dry stump height diameter with bark (*DSH*) was also measured on stump height disks for use in the calculation of the inside-bark DSH (see Compiling stem growth data below). The DSH was also used in developing models linking DSH with DBH to allow others to link our model to their studies, since most forest models use DBH as response or driving variables whereas our model used stump height ring-width data. Two radii separated by an angle of 90−180° (avoiding knots or severe reaction wood) were chosen from each disc. Visual cross-dating for each radii and core collected was conducted under a binocular microscope, and focused on the pointer years (wide and narrow rings) observed in each transect and among transects. The dated cores and radii were all carefully measured using a Velmex measuring system interfaced with the ‘Times Series Analysis Program’ (TSAP; Frank Rinntech, Heidelberg, Germany) to a precision of 0.001 mm. Visual cross-dating was verified using COFECHA [28]. The average correlation between individual series and the master chronology within each transect was well above 0.55 (P<0.01), indicating strong similarities in inter-annual ring growth pattern among trees within transects. Master chronologies between nearby transects were also well correlated (r>0.48, P<0.01), which suggests that our crossdating was reliable. Stand age for each transect was determined using the aspen stump height ring count. This may be slightly younger than the maximum stand age which can be dated from the root collar [29]. We avoided obvious multi-aged stands, nonetheless it is possible that some of our stands are older than the age of the dominant aspen [21].

### Compiling Stem Growth Data

To compute the past growth of individual trees, we averaged the two ring-width measurements corresponding to the same year for a given tree to represent annual ring growth of that tree. We then took the cumulative sum of its annual ring growth over several time intervals *t* extending back to the year of the outermost complete ring (*t* = 5, 10, 15, 20, 25, 30 and 35 years from the year of the outermost complete ring) to obtain the total radial increment in time interval *t* (*RW_t_*), for analysis. We then calculated the basal area increment (*BAI,* cm^2^) of each tree over these time intervals. To avoid approximating the pith location in the dominant tree cores, we anchored our measurements at the cambium rather than the pith. To do this we factored and substituted the cambium offset into the normal BAI equation (1) (which needs measurements from the pith), to determine BAI.
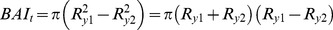
(1)

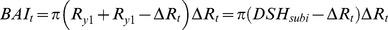
(2)


In Equations (1) and (2), we used that *R_y1_* = *DSH_subi_*/2, and *R_y2_* = *DSH_subi_*/2 −Δ*R_t_*, where Δ*R_t_  =  R_y1_–R_y2_*, *y_1_* is the calendar year of the outermost complete ring and *y_2_ = y_1_*−*t*. Lab measurements of *DSH* were converted to inside-bark DSH (*DSH_subi_*) using the published provincial equations [30] to facilitate this BAI calculation without needing the pith.

### Growth vs. Size Trend for Large and Dominant Trees

**Figure 2:**
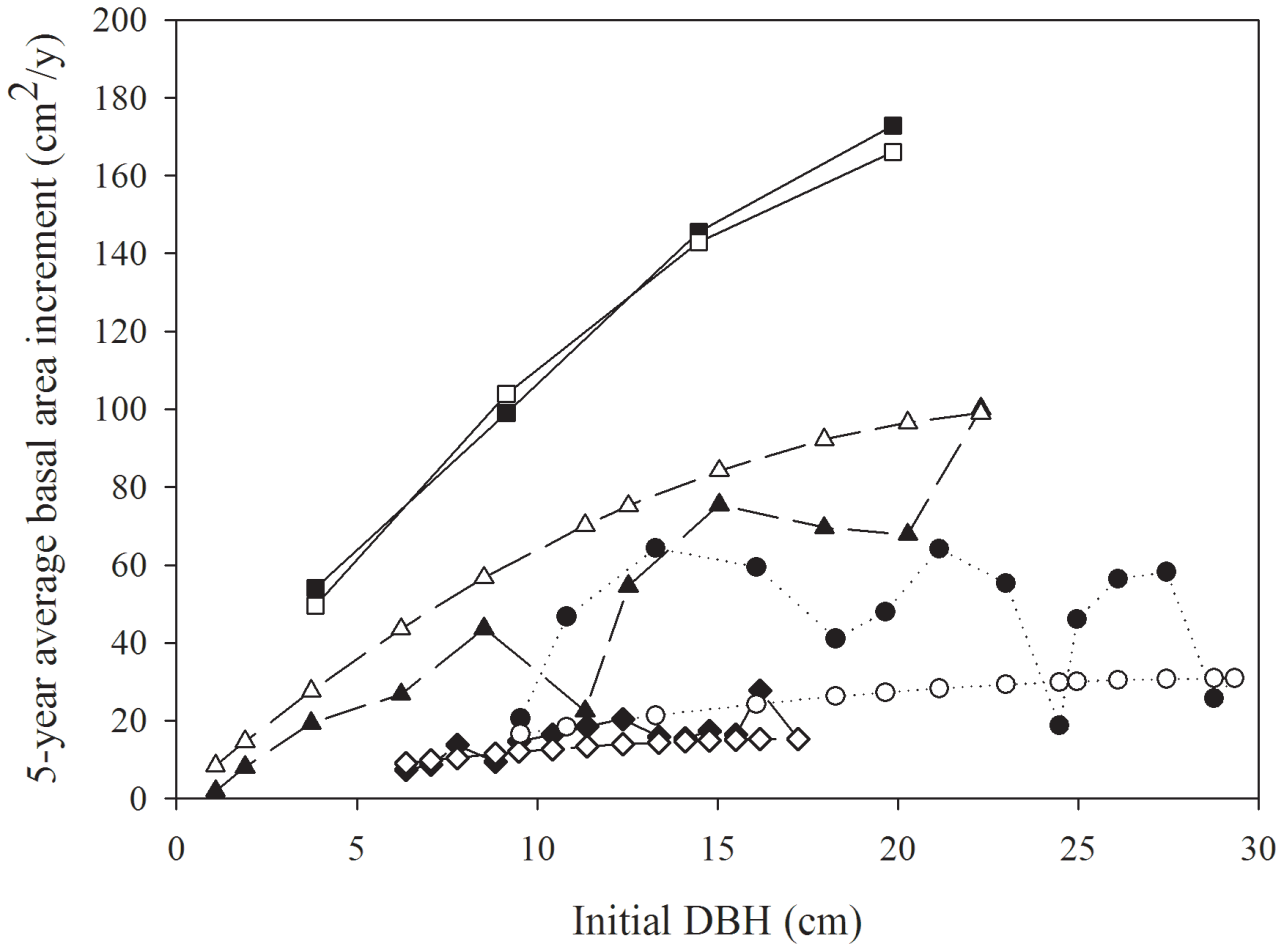
Effect of tree size (DSH) on the maximum basal area growth potential (G_L_, cm^2^/y). Data are from a random of sample large, usually dominant trees, and were calculated from the full ring chronology of these trees, from pith to bark. Each different symbol is a different tree, and the lines linking the symbols show their growth rates over time. Smooth lines (open symbols) show the modeled maximum growth potential (G_max_, Equation 3) for each tree.

Selecting large or dominant trees to identify growth potential is an effective way to condition a growth model for site, population genetics and climate effects. Additional growth reduction in suppressed trees can then be attributed to competition. However, small and very large trees generally do not have the same growth potential as intermediate trees [31]. During their early development, trees build leaf and root area, increasing their ability to acquire resources, fix carbon and increase in size. As size increases, however, maintenance respiration costs for supporting root and stem architecture also increase, and more energy is required to raise water to a taller crown, reducing photosynthetic efficiency [32]. Canham et al. [31] described this trend in radial growth using a log-normal function: the growth rate increased initially with increasing size (DBH), reached a maximum, then declined as DBH exceeded 30 to 40 cm. Others have likewise found it important to model the effect of size on growth of the subject tree, either directly [16], [33] or as part of the competition index [34]. An independent measure, such as leaf or crown surface area is desirable for assessing size-related growth potential [20], but is difficult to obtain routinely [22]. We tested Canham et al.’s [31] log-normal function, as well as quadratic, logarithmic and several other functions to model this maximum growth – size relation using the ring width sequences from the large or dominant trees (Fig. 2).

Based on these analyses, the best model to capture the growth rate vs. initial size trend for spruce and aspen was a simple parabolic function, with no linear term is given by equation (3),
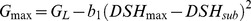
(3)where *G_max_* is the maximum potential basal area growth rate over the time interval (5, 10, etc. years), adjusted for a subject tree which is smaller than the dominant tree, *G_L_* is the average basal area growth rate of the large (usually dominant) trees, *DSH_max_* is the average diameter at stump height (0.3 m) of the large trees, *DSH_sub_* is the diameter of the subject tree, and *b_1_* is a parameter.

### Competition Indices

Distance-independent competition indices are easily obtained in the field with less cost than distance-dependent versions, but have been shown to be nearly as effective in quantifying the growth-competition relationship [22], [31], [35], [36]. There may be some scale-dependence of distance-independent indices due to the interaction of plot size and the underlying spatial pattern [37]; however, for the near-random dispersion patterns found in these forests [38], scale-dependence will be minor. We calculated six simple distance-independent competition indices for each subject tree which have been found to be effective in quantifying the growth-competition relationship [22], [34], [35]: density [N (stems/ha)], the sum of stem diameter at breast height [SDBH (m/ha)], and stand basal area [BA (m^2^/ha)]. These indices impose a series of exponent weights, *k*, on competitor size as shown in Equation (4) (*k* = 0 for N, *k = *1 for SDBH, *k = *2 for BA). Competition indices for each subject tree were calculated from plots aggregated within each transect, and were calculated using Equation (4)
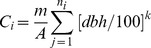
(4)where *m* is an adjustment to scale BA correctly (i.e. *m* = π/4 when *k* = 2, *m* = 1 otherwise), *A* is the sum of the plot area within each transect in ha, n_i_ is the number of trees of competitor species group *i* in the plots, *j* is the competitor tree, dbh is in cm and *k* is the size-weighting exponent. We also evaluated the same competition indices considering only trees thicker (GR) than each subject, denoted as NGR, SDBHGR, and BAGR. Indices using thicker trees assume competition is primarily one-sided, i.e. for light. Competition indices were computed for both deciduous and coniferous groups. We initially calculated indices for pine separately from the more shade-tolerant and dense-crowned spruce and occasional fir, but there were too few to obtain significant effects; we therefore redefined a conifer group which included spruce, fir, and pine.

**Figure 3:**
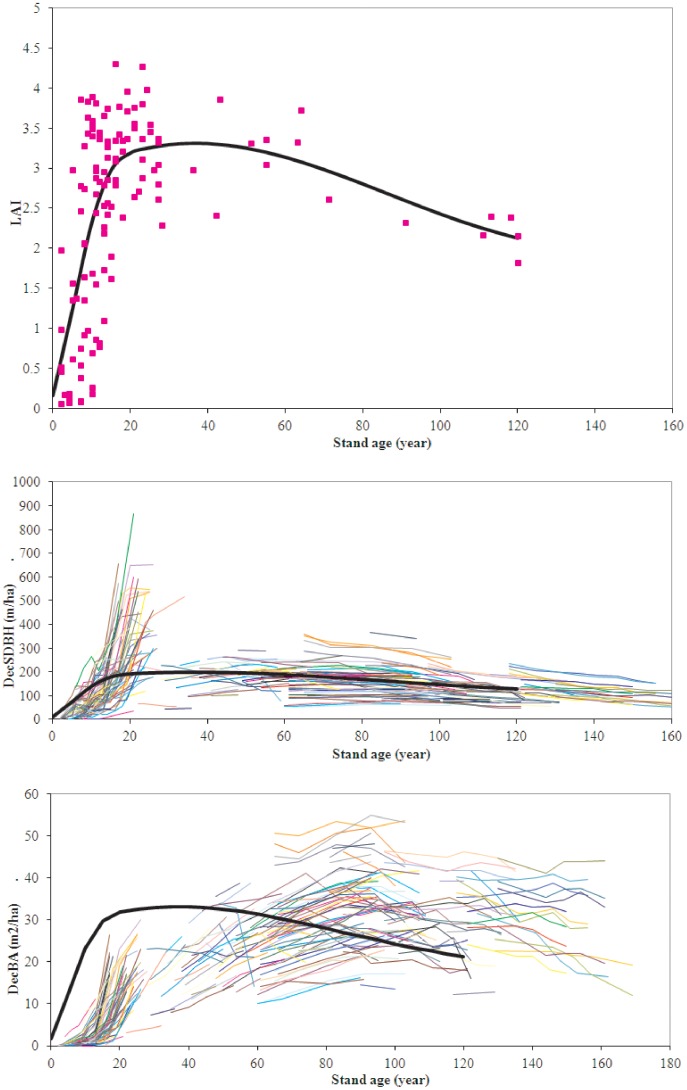
A. Leaf area index plotted against stand age for a chronosequence of pure aspen stands[6]. The dotted line is a natural cubic spline fit to this data. B. Sum of deciduous DBH per hectare (SDBH) for aspen-dominated permanent sample plots (PSPs) plotted against stand age. Each line represents the SDBH of a PSP through each of its re-measurements. These PSPs are another random sample of the same population (selected only for accessibility). The LAI vs. age trajectory is shown here too as the same dotted line, scaled to approximately fit through the middle of the PSP data. C. Basal area of deciduous trees per hectare plotted against stand age for the same PSPs as in B. The LAI vs. age dotted line is also overlaid on this data with appropriate scaling. Though it may peak more strongly in young stands, SDBH better represents the general trend of LAI vs. time for aspen stands.

There is a considerable body of literature on competition indices [20], [22], [39], most of it using empirical evidence (e.g. best RMSE or R^2^) to support the choice of a competition metric. In choosing this series of distance-independent indices, we considered the ecophysiological tenet that leaf area is generally proportional to sapwood area, which appears to hold at both a tree and stand scale [40]. We surmised that since the most conductive sapwood is laid down in the most recent growth rings [41], a good starting point would be to assume that the thickness of the sapwood stays roughly constant over time. The basal area of young trees will therefore be nearly entirely sapwood, but the ratio of sapwood basal area to total basal area will decline as the tree ages. If sapwood was an annulus of constant (and relatively small) depth, then sapwood area would scale with stem radius or DBH rather than with basal area. At a stand level then, leaf area should be proportional to the sum of DBH on a per hectare basis (SDBH). We plotted SDBH and BA against leaf area index for a chronosequence of pure aspen stands (the only boreal species for which we had such data; obtained from Lieffers et al. [6]), and found a pattern (Fig. 3) which demonstrates correspondence between SDBH and leaf area. Stand basal area, on the other hand, behaves quite differently, continuing to increase well past the 20-year age at which leaf area peaks. For aspen, we suggest SDBH should therefore be a superior long-term index of competition than basal area; we hypothesize this holds true for other tree species as well. We tested a series of indices, from density (N) through sum of DBH (SDBH) to basal area (BA) (Equation 4) for both aspen and spruce. If one index is more accurate at characterizing competition, it will be reflected in better fit statistics in the overall model (Equation 6).

We aggregated the plot competition data within transects to calculate the competition indices. Because each subject tree was randomly selected in each transect, we used the small 10 m^2^ assessment plots as samples of transect level structure. To accomplish this, we combined all competitors from all subject trees (including all subject trees except the current one), then calculated competition indices at the transect level. For the indices for thicker trees, all competitors from each transect which were thicker than the subject tree were considered. Plot area (Equation 4) was the sum of all small plot areas within the transect. Occasionally, the areas of the plots overlapped, resulting in a lack of independence among plots. However, this is roughly equivalent to sampling with replacement; the stand-level competition index values were still unbiased.

**Figure 4:**
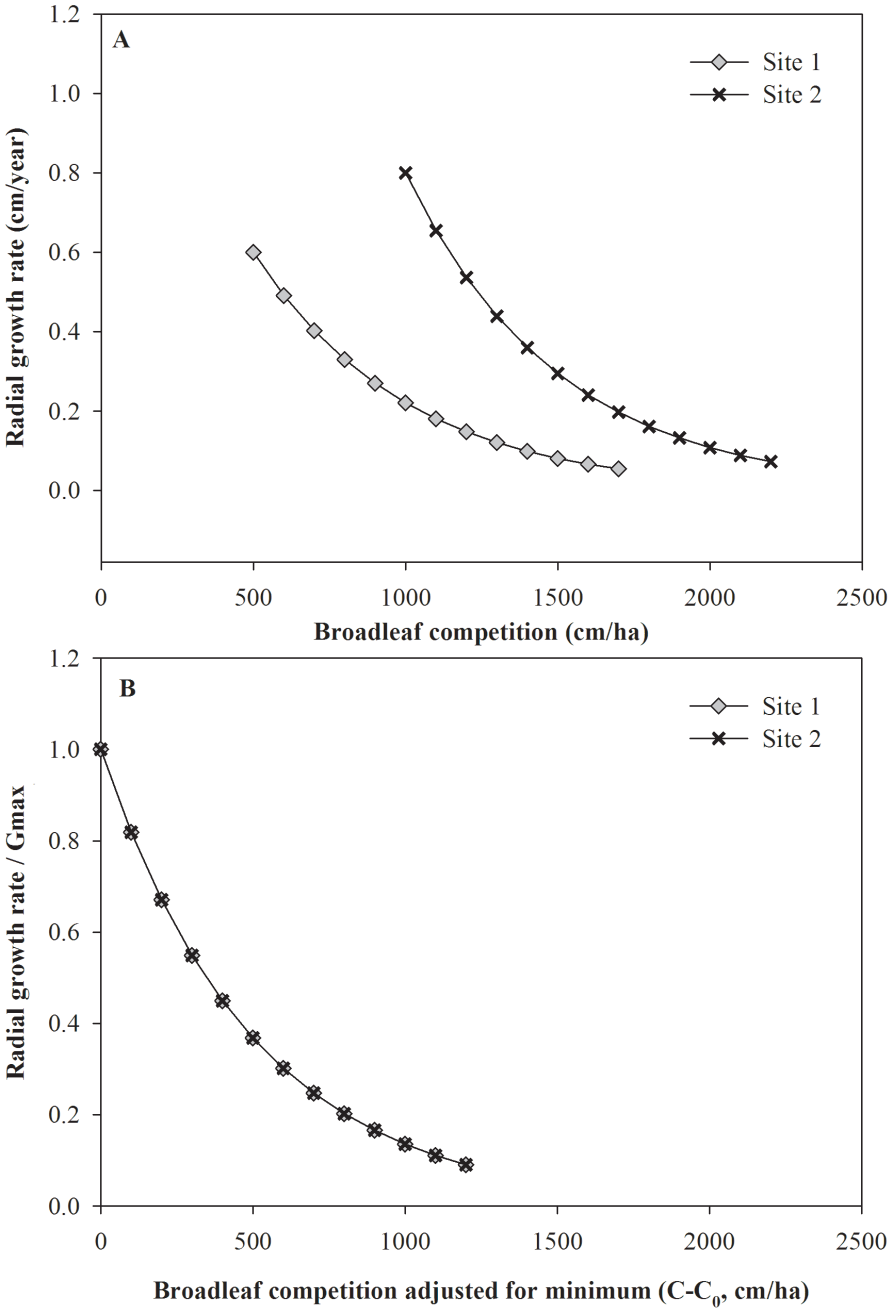
A) Hypothetical growth – competition relationship for two sites. Site 2 has a higher growth potential than site 1 but has no dominant large spruce. B) Adding the maximum growth of dominant trees to the growth response makes the growth response of both sites similar (shifts the growth response to a common maximum of one; and shifting the competition index by subtracting the competition level for the large trees found in the site moves the competition response to the left, bringing all sites onto the same growth – competition response (see Methods).



For some transects without dominant spruce nearby, we had to adjust the competition levels to recognize that our maximum spruce growth rate estimates were not competition-free. In most cases (all aspen, many spruce), we could assume the dominants are growing at their maximum potential for the site. This provides the “zero competition index” growth benchmark for the site; for the subordinate trees, this value was then reduced by the competition due to deciduous and conifer neighbours. Where there were no dominant spruce nearby, we recognized this by assessing the competition levels of these largest spruce (*C_x0_*), and used these values as an offset to properly locate these in our growth vs. competition model (Equation 5, Fig. 4).

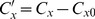
(5)Here *C*′*_x_* is the competition index for species group *x* (deciduous or coniferous) adjusted for the competition level of the largest spruce, and *C_x_* is the competition index for subject tree. For true dominants without thicker competitors, *C_x0_* = 0. This adjustment moves the growth vs competition response to the left, while the use of the growth of the largest spruce as the maximum, removes site effects and puts all trees within all sites on the same growth – competition response (Fig. 4). This worked for the competition indices for thicker trees (NGR, SDBHGR, BAGR) since these indices result in a range of competition levels for the trees in each stand, depending on their size relative to their competitors. For the other indices (N, SDBH, BA), all subordinate trees (technically this would include the large sub-dominant spruce) had the same deciduous and the same coniferous stand level competition index. Offsetting these would result in zero competition for all trees. We examined two alternatives: first, that competitors could be ignored for these large trees (consider these true dominants), and second that these few sites (six) were better left out of the data. We found little difference in parameter values or adjusted R^2^ among these alternatives, so report results for the N, SDBH and BA indices from a model which considered these large trees as dominants and assumed they had no competition (i.e. *C_x0_* = 0).

### Natural Sub-regions

In our preliminary analyses, we found no significant differences in our growth vs competition parameters (Equation 6) between Central Mixedwood and Dry Mixedwood, and between Lower Foothills and Lower Boreal Highlands forests. We therefore grouped the Central Mixedwood and Dry Mixedwood sites together as a group (CM+DM), and Lower Foothills and Lower Boreal Highlands sites together as another group (LF+LBH). These regional groupings correspond to climate patterns [23], [24]. A dummy variable was coded as 0 and 1, respectively for the CM+DM and LF+LBH, for final modeling analysis.

### Overall Model

**Figure 5:**
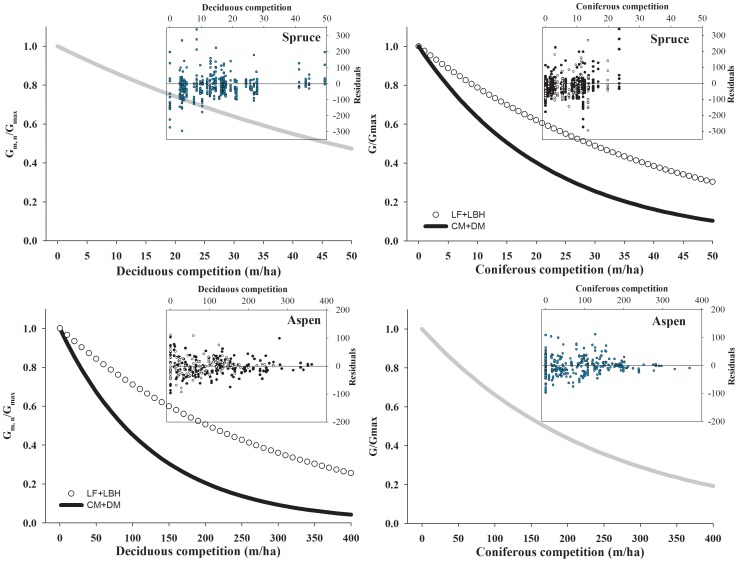
Growth ratio (G*_m_*_, *n*_/G*_max_*) vs. competition curves [BAGR for spruce (A, B), and SDBHGR for aspen (C, D)] and the residuals plots from the best nonlinear mixed model for spruce over the most recent 25 years, and for aspen over the recent 15 year (CM + DM: Central Mixedwood and Dry Mixedwood; LF+LBH: Lower Foothills and Lower Boreal Highlands).

The full model used in our analysis predicts the basal area growth of a subject tree over recent years as a function of ‘competition-free’ basal area growth from nearby dominant trees, size (*DSH*) of the nearby dominant trees and of the subject tree, deciduous and coniferous competition indices, and natural subregions (Equation 6). Basal area growth of a subject tree is given by
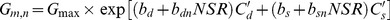
(6)where *G_m,n_* is the basal area increment of subject tree *n* in the *m*th transect over the last 5, 10, 15, 20, 25, 30, or 35 years, *G_max_* is the maximum potential basal area growth rate as above (Equation 3), *C*′*_d_* is the adjusted deciduous competition index and *C*′*_s_* is the adjusted coniferous competition index for the transect (Equation 5), *NSR* is a dummy variable (0, 1) for the two natural subregion groups, *b_d_* and *b_s_* are the parameters for deciduous and coniferous competition indices, respectively, and *b_dn_* (deciduous) and *b_sn_* (conifers) are the parameters corresponding to natural subregion effects that result in an additive shift to *b_dn_* and *b_sn_*. We fitted two of these models: one for aspen and one for spruce. Note that on Figures 4 and 5, the y-variable is *G_m_,_n_*/*G_max_*, a re-arrangement of Equation (6) which allows the transects to share a common maximum of one.

### Statistical Fitting

Since the data have a nested error structure (multiple subject trees within transects), we used nonlinear mixed modeling to fit Equation (6). Correlation within transects was modeled as a random parameter to capture this restriction on model variance. The minimum Akaike Information Criterion (AIC) [42] was used to choose the best competition index and evaluate the effects of natural subregion. For varying integration periods (from 5 years to 35 years), different dependent variables are used and therefore AIC is not a valid way to compare between models. To compare between models with differing integration periods, we instead used adjusted R^2^, a commonly used measure of goodness of fit. All analyses were conducted using the SAS nonlinear mixed modeling macro (%nlinmixed, [43]).

## Results

### Maximum Growth Model and Diameter at Stump Height: DBH Relationships

To link the maximum potential basal area increment to tree size and develop a link between dominant and subdominant tree growth potential, we developed a relationship between growth rate and size. Figure 2 demonstrates the recent 5 year basal area increment as a function of tree size (DSH) for a selection of large trees. The oscillations in basal area growth reflect the tree’s response to longer-term climate fluctuations and other factors; however there was an overall increasing and concave downward trend with increasing size. Site differences were marked, as shown by considerable variation in mean growth rate from site to site. Equation (3) effectively modeled the general increasing trend of maximum growth potential in basal area increment (BAI) vs. size. There was no significant improvement in this model from adding a linear term (P>0.05). An exponent of two was also superior to other exponents (1.5 or 2.5). To ensure that the model was biologically reasonable, a refinement was made by fixing the only fitted parameter, *b_1_*, to *G_L/_DSH_max_* ^2^, to force the function through zero when diameter of the subject tree (*DSH_sub_*) was zero and through the maximum growth when *DSH_sub_* was equal to the large tree size (*DSH_max_*). This form prevented negative growth in small trees and resulted in very little deterioration of fit. Consequently, this parameter-less version of equation (3) was used for estimating the competition-free growth potential of each subject tree as a function of its size.

We found the relationship between *DSH* and *DBH* for aspen was *DSH* = 5.30561+1.06508*DBH (Adjusted R^2^ = 0.91, P<0.0001, n = 1004) and that for spruce was *DSH* = 0.6099+1.18632*DBH (Adjusted R^2^ = 0.98, P<0.0001, n = 6913). These relationships can be used for future modeling when DBH values are required.

### Overall Growth-competition Models

When comparing growth-competition models using data integrated over the last 5, 10, 15, 20, 25, 30 and 35 years, we found the models for the past 10 to 25 years of aspen growth had the highest adjusted R^2^ values for all competition indices. The 15-year integration period was marginally higher among these, with an adjusted R^2^ of 0.667 when using SDBHGR as the predictor (Table 2; integration results with respect to the other indices are not shown). For spruce, we found the models which integrated growth over 25 or more years had the highest adjusted R^2^ with a value of 0.743 at 25 years when using BAGR as the predictor (Table 2).

**Table 2:** Parameters and the statistics of the nonlinear mixed models over the past 5, 10, 15, 20, 25, 30, and 35 years for aspen when using SDBHGR as the predictor, and for spruce when using BAGR as the predictor.

Species	Interval	Parameters (×10^−2^)				Adj.R^2^
		*b_d_*	*b_s_*	*b_dn_*	*b_sn_*	
Aspen	5	−0.434	−0.382	−0.343	0	0.573
	10	−0.383	−0.414	−0.435	0	0.665
	**15**	−**0.341**	−**0.412**	−**0.451**	**0**	**0.667**
	20	−0.323	−0.405	−0.403	0	0.662
	25	−0.300	−0.404	−0.400	0	0.622
	30	−0.299	−0.377	−0.400	0	0.544
	35	−0.263	−0.360	−0.348	0	0.554
Spruce	5	−1.338	−5.511	0	5.442	0.649
	10	−1.530	−5.442	0	4.850	0.682
	15	−1.529	−5.075	0	3.852	0.701
	20	−1.534	−4.751	0	3.013	0.721
	**25**	−**1.495**	−**4.549**	**0**	**2.160**	**0.743**
	30	−1.680	−4.051	0	0	0.742
	35	−1.824	−4.039	0	0	0.745

*Note:* See definitions for parameters in the text; Parameters that are insignificant were set to zero. Bold text shows the selected model.

We fixed the integration time at these intervals: the past 15 years for aspen and the past 25 years for spruce, to compare the six competition indices (Table 3). We found the models for aspen growth consistently perform much better in terms of AIC and adjusted R^2^ when NGR and SDBHGR were used vs. the other competition indices. Among the models for spruce growth, those which consistently performed best in terms of AIC and adjusted R^2^ used BAGR or SDBHGR as the predictor. Competition indices with all-sized trees considered (BA, SDBH, N) yielded much poorer fits than the indices which included only thicker trees.

**Table 3:** Parameters and the statistics of the nonlinear mixed models for aspen over recent 15 years and for spruce over recent 25 years when using different competition indices including sum of basal area (BA), sum of diameter at breast height (SDBH), and stem density (N), as well as similar indices for trees thicker than each subject (SDBHGR, NGR, and BAGR) as the predictor.

Species	Compet.indices	Parameters (×10^−2^)				Adj.R^2^	−2 Res loglikelihood	AIC
		*b_d_*	*b_s_*	*b_dn_*	*b_sn_*			
Aspen	BA	−3.737	0	0	0	0.404	3165.3	3169.3
	SDBH	−0.205	−0.064	−0.161	0	0.443	3178.5	3182.5
	N	−0.014	−0.007	−0.014	0	0.411	3206.0	3210.0
	BAGR	−3.709	−1.977	−3.381	0	0.547	2963.2	2967.2
	SDBHGR	−**0.341**	−**0.412**	−**0.451**	**0**	**0.667**	**2920.0**	**2924.0**
	NGR	−0.042	−0.085	−0.078	0	0.743	2905.7	2909.7
Spruce	BA	−0.901	−0.365	0	0	0.636	5815.7	5819.7
	SDBH	−0.066	−0.118	0	0.061	0.649	5828.7	5832.7
	N	0	−0.007	0	0.006	0.632	5831.7	5835.7
	BAGR	−**1.495**	−**4.549**	**0**	**2.160**	**0.743**	**5598.5**	**5602.5**
	SDBHGR	−0.123	−0.761	0	0.395	0.720	5636.5	5640.5
	NGR	−0.012	−0.108	0	0.060	0.691	5689.2	5693.2

*Note:* See definitions for parameters in the text; Parameters that are not significant were set to zero.

When we evaluated asymmetric vs. symmetric competition by testing models which simultaneously incorporated both competition indices for thicker trees and indices for all trees (e.g. BAGR and BA, SDBHGR and SDBH, or NGR and N), we found a significant but very minor improvement in fit over models with only the index in thicker trees. Under likelihood ratio tests, the additional competition index was significant for conspecifics only (i.e. SDBH of deciduous added to the aspen model, BA of conifers improved the spruce model). For aspen BAI, adjusted R^2^ increased 0.022 with both SDBH and SDBHGR in the model. For spruce BAI the increase in adjusted R^2^ from adding competitor BA to a model with competitor BAGR already in the model, was even less (0.006).

Competition effects on growth differed among natural subregion (NSR) groups. As noted in the Methods, we found the data from the Central and Dry Mixedwood (CM+DM) subregions behaved similarly, as did data from the Lower Foothills and Lower Boreal Highlands (LF+LBH). We accordingly assigned data to two natural subregion groups. Coniferous competitor effects on aspen growth did not significantly change in the two different natural subregion groups (*b_sn_* = 0); but deciduous effects on aspen growth were different (*b_dn_*≠0) (Tables 2 and 3), Conversely, the effect of conifers on spruce growth was different in the two NSR groups (parameter *b_sn_*≠0), but the effect of deciduous competition on spruce growth did not change with subregion (*b_dn_* = 0). The effect of these NSR coefficients is that, in the Central and Dry Mixedwoods, the effect of coniferous competitors, *b_s_*+*b_sn_*, was consistently greater compared to the effect of deciduous competitors, *b_d_*+*b_dn_*, on the growth of both aspen and spruce, for growth integrated over all but the shortest (5 year) integration period for aspen (Table 2). However, in the Lower Foothills and Lower Boreal Highlands, deciduous competitors had a consistently stronger effect than coniferous competitors on aspen and spruce growth (*b_d_+b_dn_*>*b_s_+b_sn_*).

As shown in Fig. 5, the effect of intra- and inter–species competition on aspen or spruce relative growth were effectively modeled as a negative exponential, with the residuals evenly distributed around zero, even for some extreme competition values. The spruce growth-coniferous competition curve is steeper in Central Mixedwood and Dry Mixedwood forests than in Lower Foothills and Lower Boreal Highlands forests (Fig. 5). For aspen, the growth response to deciduous competition is steeper in the Central and Dry Mixedwood than the Lower Foothills and Lower Boreal Highlands, whereas the aspen growth response to coniferous competition was not different between these two natural region groups.

## Discussion

In western boreal mixedwoods, modeling the long-term relationship between growth and competition has posed a challenge due to the complexity of species interactions, the high variability in growth rates, a lack of data at the critical stem exclusion phase, and the set of competition metrics which have been used. In this study, we developed a nonlinear mixed model to quantify this relationship and tested a series of simple, distance-independent competition indices. Our models demonstrated a remarkable capability to predict stem growth as a function of stem growth of the nearby dominant trees, the size of each tree relative to these dominants, intra- and interspecific competition, and ecoregion. One of the merits of our model is that we were able to model this complex growth vs site, size and competition relation through readily obtainable data, combining dendrochronological (tree-ring) sampling with simple competition indices. The model captured a large degree of the growth variance, as reflected by adjusted coefficient of determination (R^2^) values in excess of 0.67. This comprehensive model captures most of the easily described factors driving tree growth: the genetics of the population and site-level environmental factors are captured by sampling nearby dominant trees; this rate is then adjusted for tree size, then reduced by competition on an ecoregion-specific basis. Only tree-level genetic variation and damage from herbivores, pathogens and physical agents (abrasion) are not captured.

Tree-ring analysis was particularly useful for documenting the growth pattern of dominant trees. Using cross-dated basal area increment histories of the largest trees in each plot, we modeled the basal area increment as a function of initial size. As many others [16], [31] have observed, the increment of dominant trees initially increased rapidly with tree size, presumably as the trees built their crown and leaf area, then the increment increased less quickly presumably as their leaf area approached a maximum and the maintenance cost of the structural and conductive tissues increased at a greater rate than the photosynthetic supply (Fig. 2). The pattern of BAI vs. initial DSH did not exhibit the bell-shaped pattern Canham et al. [31] observed for radial increment in a sample including much larger cold-temperate trees or Huang and Titus [16] found with older boreal spruce. It is likely that BAI will also decline with size in large boreal trees; we cannot speculate since our sampling frame was young to near-mature stands. Very large trees are rare in the fire-prone boreal region.

The BAI response of dominant trees to initial size was modeled effectively by a simple function of initial DSH^2^ with fixed points at the origin and the current BAI at the most recent initial DSH (Fig. 2). Although we tested several functions with fitted parameters, this parameter-free function described the growth response of a leading dominant tree from a juvenile to a near-mature tree, with similar effectiveness. This function represents the maximum growth potential of each tree on that site over time, given the local climate in these years, physiography, soil and population-level genetics. Given that tree size may provide a surrogate measurement of tree leaf area and indirectly reflects the amount of light being absorbed and used by the tree [44], previous studies recommended including initial size of the subject tree as an important explainable variable for better explaining growth-competition relation [45], [46]. Filipescu and Comeau [35] also found a significant improvement (33%) in predictive ability of the model when initial size of the subject tree was introduced. Our model of dominant tree growth adjusted for the difference in size of the subject and dominant trees, accounted for the majority of the modeled variation in the final model, i.e., *G_max_*, determined by equation (3), was strongly correlated with the observed growth (BAI) of our aspen and of white spruce (Pearson’s r>0.75), accounting for 82–85% of the total variation (R^2^) captured in the final model (Equation 6).

It is generally assumed that height growth is a better indicator of site productivity than diameter or basal area growth, since the former is less sensitive to competition [47]. However, our work indicated that a minor amount of additional variation in dominant tree diameter growth is accounted for by stand density or basal area when fit in addition to competition indices which only consider thicker trees. This result implies that the diameter of leading trees in mid-aged aspen – spruce mixedwoods has little response to overall stand competition. This runs counter to accepted wisdom; however most studies of competition effects on height vs diameter growth have reported on the strong response of diameter (compared to height) for the average tree or with decreasing social status, not simply the response of the dominant trees. For example, the basal area increment function of Yang et al. [17] for aspen includes a competition term; however, for dominant trees (achieved by setting the social status indicators to the maximum), the growth response to a 30% shift in stand basal area is <10% change in basal area growth. Huang and Titus’ [16] spruce diameter growth function is similarly affected by a 30% change in stem density (∼10% change in growth), but nearly unaffected (<3% response) by a 30% stand basal area change. The longer crowns maintained by the dominant trees (especially spruce) likely maintain diameter growth in these primarily natural stands.

Many forest growth models are driven by site index (SI). Height vs. age and site index (the height of the dominant trees at a reference age) curves provide a simple method to model potential height growth, which is then reduced for sub-dominant trees in some individual tree models [20], [48]. The link between SI and diameter growth is less obvious; a height vs. diameter curve or allocation function [16], [49] may be used to translate height increment into a diameter increment. We did not make an explicit link to SI in this study for several reasons. SI could be obtained for leading aspen; however, current dominant white spruce were likely not dominant during much of their early growth, rendering the apparent spruce SI of questionable value as an index of spruce potential [50]. Site potential is also demonstrated in diameter growth of dominants, particularly since we found little evidence of diameter suppression in dominant trees due to stand density. By linking sub-dominant growth to leading dominant growth, our model captures the site effect without relying on SI. The competition effects which our model estimates can still be used in a SI-based model, by using Equations (4) and (6) to reduce the modeled diameter growth of the leading dominants [48].

A problem encountered in our region is that white spruce trees are usually overtopped by deciduous hardwoods early in stand development [1], [51], except in recently-established (since the 1980’s), heavily tended stands. We sought dominant spruce near our sites, but it is very likely these were overtopped earlier in their development. Thus, we cannot fully estimate the growth potential at all sizes and ages. However, when we did not find dominant spruce, we offset our white spruce response by the competition experienced by the largest white spruce sampled in or near our stands, as shown in Fig. 2. This adjustment provided mathematical consistency in the spruce growth response to competition across site differences and despite the lack of true spruce dominants on six sites. This adjustment assumes the trees respond to competition similarly and proportionally to the maximum potential (*G_max_*) across all stands (though we did allow for differences in competition response between ecoregions). Since it is almost universal in mixed forests that the potential competition-free growth of the slower-growing species is unknown, but that competition levels and growth rates can be measured, the reconciliation via Equation (5), and illustrated in Fig. 2, is a significant contribution to modeling competition in mixed forests.

Development, discussion and comparisons of indices for competition have been the subject of considerable work [22]. Burton [39] noted a failure of competition indices is that they are generally static snapshots for one stand type at one age. Since we intend this work to support a long-term forest growth model for pure and mixed stands [48], it was important to find an index that could capture the change in competitive dynamics with stand age. We argue in our Methods, that an index summing diameter (SDBH and SDBHGR) should be more effective than one which sums DBH^2^, such as the more commonly used basal area (BA or BAGR). We note that SDBH approximates a chronosequence of aspen stand leaf area much more effectively than basal area (Fig. 3). As an index for the 20–80 year-old stands sampled in this study, SDBH and SDBHGR were both more effective (in terms of improved adjusted R^2^ and reduced AIC) than BA and BAGR in a growth vs. competition model for aspen. For spruce, SDBHGR was also very effective, though slightly inferior to BAGR. Aspen leaf area (and SDBH) have peaked and are declining in the age range we sampled, while these spruce variables have not, which may be why SDBH and BA or SDBHGR and BAGR show similar effectiveness for spruce. The smaller size of the spruce relative to the aspen may also be a factor.

Interestingly, the density of thicker trees (NGR) was a superior index for aspen growth. Newsome et al. [52] also reported the effectiveness of NGR in pine - aspen stands in British Columbia. However, further analysis [53] has indicated that NGR was less effective for describing variation in height and diameter growth of lodgepole pine than other competition indices which incorporate competitor diameter. During the stem exclusion and understory reinitiation stages in these aspen dominated stands, size and density are strongly coupled through self-thinning dynamics. Therefore, it may not be necessary to include both in a competition index applied to these natural stands. However, there are many silvicultural treatments being applied in the western boreal which strongly alter aspen density, and would lead to uncoupling of the size – density relationship. Since this model will be applied to such stands, we feel SDBHGR or BAGR are more robust indices, but nonetheless report all models (Table 3).

In terms of species’ competitive effects, our modeling results showed that spruce competition had a greater effect than deciduous competition on aspen and white spruce growth in Central and Dry Mixedwood ecoregions, as indicated by *b_s_* being more negative than *b_d_*. This is in agreement with Stadt et al. [22] who compared the competitive effect of the five dominant boreal species using PSP data and found white spruce caused larger growth reductions than trembling aspen competition. However, this relation was reversed in the Lower Foothills and Lower Boreal Highlands, with aspen having a stronger competitive effect than spruce. This may be a consequence of the lack of large spruce in our stands in these ecoregions; we may not have obtained sites with enough large spruce in these natural stands to show any strong effect on aspen growth. However, regional differences are common. Wright et al. [10] and Filipescu and Comeau [33], for example, found different growth vs. light competition relationships among regions. This appears to be a complex interaction: regional differences are primarily climatic, and climate effects on spruce growth can be exacerbated by deciduous competition [54].

We acknowledge that while our growth rate sample is retrospective, our competition sample is current. Metasaranta and Lieffers [15] reconstructed past competition levels in small pure pine plots by dendrochronological analysis on all trees (live and dead) within a plot. We were unable to achieve this in our plots due to the rapid decomposition of the broadleaf species, preventing cross-dating of their past size and death year.

Another important finding is that current estimates of competition can be linked to long-term growth, i.e. 10–25 years for aspen and >25 years for spruce. Commonly, growth is determined for a short period, related to the PSP measurement interval or a few recent ring widths [10], [15], [33], [35]. Our study demonstrates that current competition is more strongly related to stem growth over the past 10 to 25 years vs. shorter periods. Our use of stable competition indices may be part of the reason. Longer periods may smooth out noise in the growth of both subject trees and competitors, especially if the competitive position of subdominant trees remains the same throughout this time.

## Conclusion

We used tree ring analysis as a substitute for permanent sample plots to obtain data for quantifying the effect of inter- and intra-species competition on stem growth of the two predominant species, white spruce and trembling aspen in the boreal mixedwood forest of western Canada. Through a nonlinear mixed modeling approach, we established a powerful model to predict basal area growth as the function of the growth of dominant trees, the difference in size between the nearby dominant trees and of subject trees, deciduous and coniferous competition levels, and ecoregions. We found the competition index using the sum of diameter at breast height of thicker trees is a reasonable and ecologically meaningful predictor for predicting aspen and white spruce growth, though other indices also fit well.
